# Pastoral Farming Systems in Arid Regions: Typology of Small Ruminant Farms in Southern Tunisia

**DOI:** 10.3390/ani16060902

**Published:** 2026-03-13

**Authors:** Aicha Laroussi, Daniel Martin-Collado, Ahlem Atoui, Roukaya Chibani, Farah Ben Salem, Mouldi Abdennebi, Lamia Doghbri, Mohamed Jaouad, Sghaier Najari

**Affiliations:** 1 IRA, Laboratory of Livestock and Wildlife, Institute of Arid Regions, Medenine 4119, Tunisianajarisghaier@yahoo.fr (S.N.); 2 Faculty of Sciences of Gabès (FSG), University of Gabes, Cité Erriadh, Zrig, Gabès 6072, Tunisia; 3 Centro de Investigación y Tecnología Agroalimentaria de Aragón, Avda. Montañana 930, 50059 Zaragoza, Spain

**Keywords:** pastoral farming systems, typology, rangeland management, arid zones, small ruminants, camels, southern Tunisia

## Abstract

This study investigates the typology of the pastoral farming systems in the arid region of southern Tunisia, with a particular focus on the governorate of Tataouine. A field survey was conducted among 111 livestock farmers distributed across different agro-ecological zones. The typology of breeding systems was established using a Factor Analysis of Mixed Data (FAMD), which identified eleven dimensions explaining 69.74% of the total data variance. The first three dimensions accounted for 15.91%, 8.79%, and 7.67% of the variability, respectively, and were defined by herd composition, resource availability, and management strategies, including variables such as the number of goats, sheep, and camels, distance to water sources, infrastructure, reproductive practices, and workforce availability. Hierarchical clustering revealed three distinct systems: System 1, regrouping “Small Urban Farmers”, defined by small-scale operations relying on family labor, localized feed resources, and market-driven production targeting urban consumers; System 2, representing large livestock, composed of professionalized operations with improved infrastructure, hired labor, and transhumance practices to optimize resource use and productivity; and System 3, for herds with camels, characterized by extensive systems utilizing collective rangelands and camels to adapt to arid conditions and ensure ecological resilience. The results emphasize how ecological constraints, infrastructure, and spatial organization shape the diversity of these systems. This typology provides critical insights into the challenges and potential of livestock farming in arid environments and offers a foundation for designing targeted interventions to support the sustainability of pastoral systems under increasing environmental and economic pressures.

## Simple Summary

Livestock farming in arid regions is under increasing pressure due to water scarcity, degradation of grazing lands, and rising production costs. In southern Tunisia, sheep, goats, and camels remain essential for rural livelihoods, but pastoral farming systems are changing rapidly. This study aimed to identify and describe the main types of pastoral farming systems currently operating in this region. We surveyed livestock farmers in the Tataouine area and examined their herd size, animal species, access to water and grazing land, feeding practices, labor organization, and animal management. The analysis revealed three main farming systems: small farms located near urban areas with limited herd sizes and strong dependence on markets; larger farms with more organized labor and mixed use of grazing and purchased feed; and extensive systems combining small ruminants and camels, relying mainly on natural rangelands and seasonal movement. Each system shows different levels of vulnerability and resilience to environmental and economic constraints. Identifying these farming systems helps improve our understanding of how livestock farmers adapt to harsh conditions and provides valuable guidance for developing targeted policies to support sustainable livestock production and rural livelihoods in arid environments.

## 1. Introduction

Arid and semi-arid regions cover over 40% of the world’s land surface and are home to approximately 2.1 billion people, many of whom depend directly on pastoralism for their livelihoods [1]. Small ruminant farming, particularly of sheep and goats, is a key component of these pastoral systems due to their adaptability to harsh climatic conditions, contributing to food security and income stability [2]. However, climate change, desertification, and socio-economic transformations are reshaping these farming systems. In North Africa, pastoral systems have progressively evolved into agro-pastoral models that integrate crop cultivation with livestock farming. This transition has been largely driven by agricultural intensification, shifting market demands, and environmental constraints [3,4].

Small ruminant production plays a crucial economic role in arid regions, as it offers a relatively high return on minimal investment, particularly in arid environments. This is due to the low input needs and the relatively high reproductive performance of sheep and goats [4]. Contrary to other world regions, small ruminants are still of great economic and social importance in marginal and arid regions of North Africa, and this importance is growing. In Tunisia, for instance, the sheep population grew from 8.1 million in 1990 to 8.9 million in 2023, while the goat population increased from 1.2 million to nearly 1.5 million over the same period [5]. However, similarly to other regions, pastoral systems in Tunisia face growing challenges, including natural resource degradation, demographic pressures, and changing land use patterns, which threaten their long-term sustainability [6,7]. In response, many livestock farmers have adjusted their flock structures, increasingly favouring sheep over mixed flocks, while also adopting more intensive feeding strategies to compensate for declining pasture availability [8].

Previous studies have developed farm typologies in various parts of North Africa, highlighting the diversity of livestock production systems and their adaptation to environmental and socio-economic constraints [9,10]. In Tunisia, farm classification approaches have also been applied to livestock systems, particularly in arid and semi-arid regions, to characterize herd structure, mobility, and resource/constraints management strategies. Several authors have documented the ongoing transformation of pastoral farming systems in North Africa. For instance, ref. [11] emphasized the shift from traditional nomadic systems to more integrated agro-pastoral models in response to land degradation and drought pressures. Similarly, ref. [8] observed a marked reduction in transhumance practices among southern Tunisian herders, who increasingly rely on localized grazing and market-purchased feed resources. Beyond southern Tunisia, the axes that structure our typology include herd composition and scale, access to resources and infrastructure (water, rangelands, livestock housing facilities), and organization/mobility (labor, prophylaxis, transhumance). These axes recur across other drylands. In the Algerian steppe zones adjacent to our study area, recent evaluations linking typology and sustainability distinguish systems based on dependence on external inputs versus rangeland resources and on management mobility versus sedentarization, consistent with the gradients identified in our typology [12]. In Egypt’s desert context, analyses of livelihood strategies under drought highlight the central roles of livestock and mobility in adaptation, consistent with our FAMD components [11]. At other latitudes, Patagonia illustrates the seasonal constraint (insufficient winter rangelands) that necessitates targeted supplementation in small ruminants, a mechanism analogous to that observed among our ‘small peri-urban’ farms [13]. Finally, in Central Asia, the efficiency of professionalized systems remains strongly conditioned by land-tenure governance, differentiated access to pastures, and mobility [14]. Taken together, these studies indicate that the determinants we identify are generic to arid and semi-arid zones, supporting the transferability of our typology.

Considering these substantial shifts, the present study aims to deliver an updated classification of pastoral farming systems in southern Tunisia, capturing the diversity of current practices, structural forms, and adaptation strategies in an increasingly constrained environment.

This study aims to refine the classification of pastoral farming systems in southern Tunisia by considering the socio-economic and technical changes that have reshaped the sector in recent years rather than the climate change affecting the irregularity of natural resources. By identifying emerging patterns of flock composition, resource use, and adaptation practices, this typology provides insights of current farm types present in pastoral systems. Ultimately, this study not only updates the scientific understanding of pastoral transformations in Tunisia but also supports broader efforts to build resilient, sustainable rural livelihoods across the arid zones of the Mediterranean.

## 2. Materials and Methods

### 2.1. Description of the Study Area

**Figure 1:**
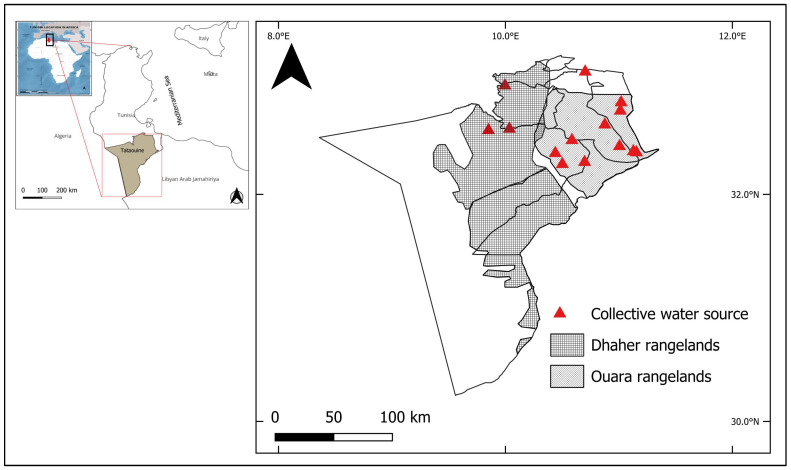
Study area.

The study was conducted in the arid regions of the Tataouine governorate in southeastern Tunisia, as shown in Figure 1. The area includes both remote rangelands and peri-urban pastoral zones located near towns and settlements. Rangelands cover El Ouara and Dhahar pastures located respectively in the east and south of the governorate. The peri-urban zones are essential to understanding the dynamics of small-scale pastoral farming systems. The rangelands extend over more than 1.5 million hectares and are representative of typical arid grazing areas in Tunisia. The climate of El Ouara is classified as a hot desert climate (Köppen classification). Similarly, the Dhahar region experiences an arid climate with low and irregular rainfall, averaging about 15–20 rainy days per year. Annual temperatures range around 19 °C, with winter lows of approximately 8 °C. These climatic conditions directly impact forage availability and the organization of pastoral practices, as the limited and irregular rainfall constrains natural pasture growth, forcing farmers to rely on transhumance, feed supplementation, or reduced flock sizes to cope with seasonal shortages [8]. The irregular rainfall also makes collective water sources a precious resource in the region that determines the movements of flocks. In such dryland systems, ensuring year-round access to water is often more critical than access to forage. In 2019, the livestock population in the Tataouine region included approximately 1,200,000 sheep, 350,000 goats, and 90,000 camels [15].

### 2.2. Data Collection

Data were collected using a face-to-face farmer survey from July to December 2020. The questionnaire aimed to capture the structure and management of different pastoral farms in the area under study. It was divided into five parts: general farm characteristics, flock management, flock size and structure, natural resources and infrastructure, feeding practices and reproduction and health. Farmers were not selected in advance; instead, all livestock keepers encountered during the field survey were interviewed without prior sampling. Initially, data collection involved 136 farmers, but only 111 surveys were retained for analysis after excluding incomplete responses.

### 2.3. Data Analysis

#### 2.3.1. Selection of Variables

We first conducted a univariate analysis to examine the distribution and statistical parameters of all variables collected through the questionnaire. Variables showing little or no variability among farmers were excluded from further analysis, as they offered limited discriminatory power. From the remaining set, thirty-four variables were selected based on their ability to describe the structural and functional heterogeneity of pastoral farms in the study area. These variables were chosen for their relevance to characterizing flock composition, management practices, access to natural resources, reproduction strategies, and production objectives. Table 1 presents the selected variables along with their types, modalities or units, and descriptive statistics.

#### 2.3.2. Factor Analysis and Clustering

The study uses a multivariate approach (FAMD-HCPC) to integrate both categorical and quantitative farm-level data, which is still rarely applied to pastoral contexts in North Africa [3]. The application of FAMD-HCPC in this context allows for the empirical identification of farm typologies in socio-ecological systems characterized by complex, multi-level interactions [3]. We conducted a Factor Analysis of Mixed Data (FAMD) followed by Hierarchical Clustering on Principal Components (HCPC). FAMD is particularly suitable for datasets containing both categorical and numerical variables and it enables the extraction of key principal components [16]. We included 29 variables in the analysis.

The number of dimensions retained for clustering was based on the Kaiser criterion (eigenvalues > 1), visual inspection of the scree plot, and the cumulative percentage of explained variance. A total of 11 dimensions, explaining 69.74% of the total variance, were selected as they represented a good balance between dimensionality reduction and information retention. These components were then used as input for the HCPC, which combines hierarchical clustering and k-means consolidation to identify stable and well-separated farm clusters.

Clustering analysis is a classification technique that groups individuals (here farms) into distinct clusters (here typologies), maximizing intra-cluster similarity while ensuring inter-cluster divergence [17] (Frades and Matthiesen, 2010). The use of HCPC allows for the formation of nested groups, providing a structured classification of individuals, and is often considered more robust than other clustering techniques [18].

The analysis was conducted in R software (version 4.4.1, R Foundation for Statistical Computing) using FactoMineR (4.4.1) and factoextra packages (4.4.1). The calculation method combines hierarchical agglomerative clustering with k-means clustering based on the factor analysis output. The hierarchical clustering tree was constructed using Ward’s method, which minimizes total variance within clusters, and the Euclidean distance matrix to assess similarities between individuals. Afterwards, k-means consolidation was performed to refine the cluster boundaries. The optimal number of clusters was determined based on inertia gain values and supported by the majority of clustering indices provided by the NbClust package (3.0.1). This selection ensured that the final cluster groups captured the most relevant variability of the farms.

#### 2.3.3. Characterization of Farming Systems

Once the clusters were identified, we compared the farms belonging to different clusters to assess the differences between farm typologies. For quantitative variables, we used the Kruskal–Wallis test followed by Dunn’s post hoc test (with Bonferroni correction); for qualitative variables, we used the Chi-square test. A *p*-value threshold of 0.05 was used to determine statistical significance.

## 3. Results

The FAMD helped reduce dimensionality and highlight key structural and functional variables explaining the diversity of farms. Based on this multivariate structure, the HCPC identified three distinct farm typologies. The present section provides a detailed profile of each typology, focusing on herd composition, labor organization, feeding and reproductive strategies, and access to natural resources.

### 3.1. Principal Components and Farm Structure (FAMD Analysis)

We interpreted the contribution of the variables to the first three dimensions identified through FAMD (Table 2). This highlighted the most influential factors shaping the structure of farms in the study area.

Dimension 1 was primarily defined by the total number of goats (0.70), total number of sheep (0.60), and number of camels (0.39). This dimension reflects differences in flock size and species composition, acting as a proxy for overall farm scale and structure. These variables distinguish diverse farms with several species from more specialized farms.

Dimension 2 was characterized by variables related to resource access and infrastructure, including the distance to water sources (0.44), availability of farm infrastructure (0.35), and the use of feed supplements (0.31). This dimension differentiates farms based on the type of water source (collective or private), the infrastructure available (fixed, mobile or building) and the use of concentrate or not on farms.

Dimension 3 highlighted aspects of reproductive and human resource management, with key contributions from reproductive strategy (0.49), water-related constraints (0.36), and workforce type (0.27). This dimension differentiates farms based on their reproductive control (controlled vs. uncontrolled), exposure to water access problems, and reliance on family or hired labor.

### 3.2. Typology of Pastoral Farming Systems

**Figure 2:**
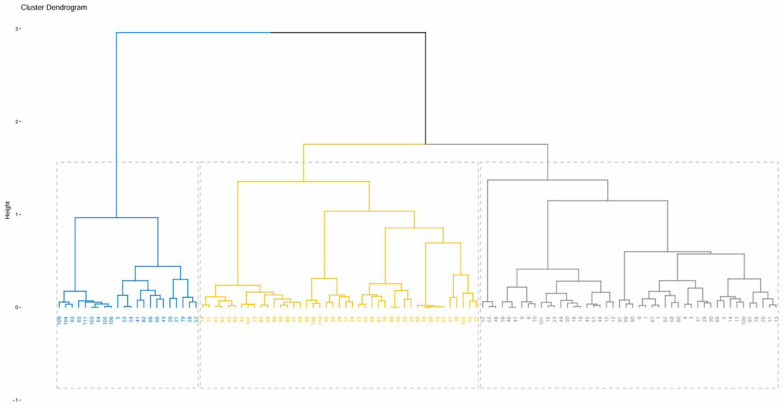
Dendrogram of the Hierarchical Clustering on Principal Components (HCPC) showing the three clusters of pastoral systems, with three different colors.

The HCPC revealed three clusters, i.e., three types of pastoral systems. These clusters are illustrated in Figure 2, which presents the dendrogram resulting from the hierarchical classification. Table 3 and Table 4 show the percentage of responses for the categorical and numerical variables within each cluster.

System 1: Small Urban Farmers

This system gathers 35 farms, primarily located in peri-urban areas. For a majority of farmers (54.3%), livestock farming was their main activity. Labor was mostly based on family members (71.4%), with little recourse to the external workforce. Herd sizes were modest, with an average of 171 sheep, 49 goats, and only 3 camels. Feeding strategies show a strong reliance on wheat bran (97%) and olive pomace (69%), whereas only 37% of farmers use concentrates. Reproductive management was mostly uncontrolled (77%), with limited adoption of the male effect (17%). Access to water was facilitated by private wells (80%), and 60% of the farmers report implementing feed separation. However, parasite-related health issues are commonly reported (54%). Animal sales were generally driven by market availability (74%) and commercial motivations (57%).

System 2: Large Farms

This group comprises 38 farms, typically situated in rural zones. Only 44.7% of farmers identify livestock as their main activity, suggesting a partial reliance on other income sources. Labor organization was more professionalized, with 71.1% of farms depending on hired workers. Flock sizes were moderate, with averages of 330 sheep, 126 goats, and 6 camels. Feeding practices rely significantly on wheat bran (71%), while the use of concentrates is very limited (3%). Reproductive management remains largely uncontrolled (74%), and only 39% of farmers report using the male effect. Most farms (82%) use communal water sources, and 74% are located within 10 km of water access. Farm infrastructure is relatively developed, with 63% using fixed buildings and 32% using transportable infrastructure. Notably, 79% of farmers implement prophylactic health plans, and transhumance is practiced by 71% of them.

System 3: Large Farms with camels

This system included 38 farms, generally situated in more remote, extensive grazing zones. Only for 28.9% of farmers was livestock their primary activity. The workforce was mixed, with 47.4% relying on family members, 31.6% on hired labor, and 21.1% combining both. Animal numbers were the highest among the systems, averaging 446 sheep, 83 goats, and 23 camels. Feeding strategies reflected a strong dependence on collective rangelands (97%), with limited use of wheat bran (45%), olive pomace (11%), and concentrates (11%). Reproductive management is more structured, with 68% of farmers adopting controlled strategies and 74% using the male effect. Water is primarily sourced from communal wells (95%), and access is more constrained, with 39% of farms located over 10 km from water points. Transhumance is highly prevalent (87%), and 92% of farmers separate feed for different animal groups.

## 4. Discussion

### 4.1. Interpretation of the Three Farming Systems

Our analysis highlights three clearly differentiated pastoral farming systems in southern Tunisia’s arid zones, more precisely in the Tataouine Governorate. In this paper, “pastoral farming systems” are defined as livestock-based production units in arid and semi-arid settings that rely primarily on natural rangelands (collective and/or private), seasonal mobility (transhumance), and adaptive management of key inputs (water, feed supplementation, reproduction), notably for small ruminants and camels. Although these systems share a harsh and irregular environment, they reflect diverse adaptation strategies shaped by technical choices, resource availability, and unequal access to institutional support. This discussion is structured as follows: we first explore the defining characteristics, strengths, and weaknesses of each system type; second, we discuss broader challenges to resilience; third, we analyze the human and institutional dimensions; and finally, we provide actionable recommendations for development.

System 1 Small Urban Farmers: Market Proximity with Structural Vulnerabilities: This group includes small-scale pastoral farming systems located near urban centers. Their proximity to towns offers additional feed resources and distinct market advantages, including shorter supply chains, reduced transport costs, and a direct outlet for meat and dairy. However, these benefits coexist with structural constraints: limited herd sizes, reliance on family labor, and a heavy dependence on low-cost feed like crop residues. While affordable, these feeds lack nutritional quality, especially during dry periods, exposing herds to undernutrition and reducing productivity. The use of communal water sources, often poorly managed, adds further vulnerability, increasing the risk of water shortages and contamination. These constraints are compounded by fixed housing structures that promote the accumulation of pathogens, elevated stocking densities, and the lack of institutional support for animal health services. Farmers are often expected to cover veterinary costs entirely out of pocket, and public subsidies or support programs are frequently unavailable even in zones near administrative centers. These limitations mirror findings by [19] and [20], who noted similar vulnerabilities in peri-urban pastoral systems. Overall, while this typology benefits from market access, it remains structurally fragile and environmentally vulnerable.

System 2 Large farms Technical Efficiency with Economic Risks: These farms reflect a higher level of professionalization, with improved infrastructure, salaried labor, and organized health programs. Feeding is based on both purchased inputs (bran, concentrates) and crop residues, enabling stable production year-round. However, this feeding strategy creates dependency on external markets, making farms sensitive to price spikes and input shortages. This trade-off—technical efficiency versus economic exposure is well documented in dryland pastoral farming systems [19]. Transhumance remains a strategic asset for these farms, offering access to seasonal rangelands and reducing feed costs during dry spells. Yet this mobility depends on maintaining grazing routes and land tenure security, which are increasingly challenged by land fragmentation. While Type 2 farms are resilient in terms of productivity and animal health, they remain economically vulnerable, especially in volatile markets. Moreover, the growing desertification threats risk seriously reducing the rangelands’ ability to serve these herders.

System 3 Large farms with Camels: Ecological Robustness, Institutional Isolation: These farms operate across vast rangelands, relying on extensive grazing and integrating camels alongside small ruminants. Their low input system—minimal use of commercial feeds and infrastructure—reduces market dependence and enhances resilience to economic shocks. Camels, in particular, offer unique advantages: their ability to forage in arid environments, survive water scarcity, and access ecological niches where other species cannot thrive. These findings align with [8] and [21], who emphasize the multifunctional role of camels in desert ecosystems.

However, their geographic remoteness hinders access to veterinary services and health programs, exposing herds to disease risks. Water access remains fragile due to reliance on shared wells and increasing competition. Although transhumance is widely practiced in this group, its viability is threatened by shrinking communal rangelands. These systems also face institutional marginalization, with limited integration into cooperatives or development programs. Despite their ecological strengths, Type 3 farms remain under-supported. Notably, ref. [8] showed that when properly managed, camel-based systems can achieve profitability comparable to intensive sheep farms, highlighting their economic potential if given the right support.

The dynamics highlighted by our typology align with regional trends and strengthen its transferability: in the Algerian steppe, the differentiation of systems by access to rangelands and water and by the level of supplementation confirms our “resource–input” and “mobility–sedentarization” gradients [12]; in southern Tunisia, intensification via fodder crops and purchased feeds at the expense of natural rangelands follows the trajectory we observe [8]; in Morocco, camel specialization supports both economic and ecological resilience [22]. These findings are consistent with other arid zones of the Maghreb: in the Tunisian semi-arid zone, three sheep systems differ in their reliance on irrigation, fodder crops, and concentrates [23]; in the Adrar oases (Algeria), an integrated crop–livestock model using local by-products shows analogies with our “large camel-based farms” cluster [24]. Overall, variation in input dependence and diversification of livestock types appear generic to arid environments, which justifies transferable though locally adapted recommendations on rational resource management, support for the camel value chain, and optimization of input use.

### 4.2. Resilience Challenges and Broader Implications

Our findings align with recent research showing how arid pastoral farming systems are shifting under environmental and market pressure. Ref. [8] observed that over a 15-year period, most farms in southern Tunisia expanded cereal cultivation and feed supplementation while reducing reliance on natural grazing. While this strategy boosted output, it also increased vulnerability to feed market fluctuations, as observed in our study. The move toward intensification, without structural support (land rights, infrastructure), risks long-term instability [25]), reinforcing this concern by showing that efforts to intensify production in North African drylands often lead to increased input dependency and ecological stress, unless accompanied by institutional and territorial planning. Their findings emphasize the need to avoid isolated technical fixes and instead promote systemic, place-based strategies.

These findings confirm that pastoral transformations in southern Tunisia, marked by reduced herd mobility, growing reliance on external feed, and institutional asymmetries, mirror broader dynamics observed across the arid Mediterranean. As demonstrated by [11] and [25], the resilience of pastoral systems increasingly depends on their capacity to adapt technically and socially, while maintaining ecological functionality. Our updated typology highlights these trade-offs and supports the design of localized policies that promote both livelihood stability and sustainable resource use in dryland contexts.

Goats continue to play a central role in these systems due to their resilience. As [26] observed, goats are better suited than sheep or cattle to withstand heat stress, poor forage, and water scarcity in southern Tunisia. However, climate change is intensifying these pressures. A growing number of farmers now report difficulties in accessing water and a clear degradation of natural grazing areas, underlining the ecological strain facing these systems. Ref. [26] further quantified these effects, showing that each additional degree of extreme heat can reduce kids’ weight gain by up to 450 g, underscoring the urgency of climate-resilient genetics and adaptive management.

### 4.3. Human Dimension and Implications for Public Policy

Adaptation is not only technical it is social. Farmers’ ability to adopt new practices depends on education, access to extension, and the strength of their community networks. Studies by [27] and [28] found that farmer training, demonstrations, and peer learning greatly boost innovation uptake in Tunisia’s drylands. Similarly, in our observations, communities with active associations or regular contact with extension services were more likely to adopt drought-resilient strategies. Conversely, remote areas lacking institutional support often remain locked in traditional practices. These findings emphasize that strengthening local organizations, cooperatives, producer groups, and water user associations is just as vital as introducing new technology. Beyond structural differences between farm types, gender also plays a key role in the functioning of these pastoral systems. Ref. [25] showed that women represent 49% of dairy processors in Tataouine and that women-led groups can substantially increase the value of goat milk through artisanal cheeses and butter. Although our survey did not explicitly stratify farms by gender roles, the prevalence of family labour in Types 1 and 3 suggests that women’s contribution to milking and processing is likely to be important. Future work should therefore combine farm typology with a gender-disaggregated analysis of labour and decision-making to better design inclusive interventions (support women’s cooperatives in peri-urban systems).

Our three types have functional counterparts in other drylands: peri-urban smallholder farms combine market proximity with dependence on purchased feeds/by products, a configuration reported in the Algerian steppe (access to inputs and water as key performance drivers) and, by seasonal analogy, in Patagonia, where insufficient winter rangelands necessitate targeted supplementation [12,13]; large, professionalized farms (infrastructure, salaried labor, herd-health plans, seasonal mobility) align with agropastoral intensification trajectories in arid areas, while in Central Asia performance hinges on land tenure security and the governance of pastures/markets [11,14]; camel-based extensive systems capitalize on collective rangelands and the plasticity of the dromedary; in Morocco, their development contributes to households’ economic and ecological resilience [22]. Consequently, the policy levers proposed for Tataouine water and health service provision and basic facilities (type 1), risk management and pastoral governance (type 2), veterinary support, strategic watering points, and camel value-chain development (type3) are transferable, with local adaptation, to the North African steppes and Sahelian savannas, where similar constraints (forage irregularity, water scarcity, distances, input price volatility, land-tenure security) structure system diversity [8,29].

Beyond the local context of the Tataouine governorate, these findings provide transferable insights into the structural drivers of livestock system resilience in arid and semi-arid regions globally. Our results highlight that while the fundamental architecture of pastoral systems—particularly in areas like El Ouara and Dhahar—remains relatively stable, their survival is increasingly dependent on the evolution of functional adaptation strategies rather than structural changes. This ‘strategic fluidity,’ characterized by diversified herd management, tactical mobility, and adaptive resource use under climatic and socio-economic constraints, offers evidence-based elements to inform resilience-oriented policies in comparable dryland contexts worldwide. Consequently, understanding these internal dynamics is crucial for developing sustainable livestock policies that respond to environmental variability and global policy pressures.

## 5. Conclusions

This study identified three distinct pastoral farming systems in southern Tunisia, each shaped by specific structural characteristics, resource access, and management practices. From market-oriented smallholder farms near urban centers to large-scale camel-based systems adapted to extensive rangelands, the diversity observed reflects the range of strategies used by herders to sustain their activity in arid conditions. These differences reveal contrasting levels of resilience and vulnerability—economic dependency in large farms, institutional neglect in extensive systems, and infrastructural constraints in peri-urban areas. Recognizing this diversity is essential to inform context-specific interventions. Strengthening veterinary support, improving access to water and forage, and reinforcing collective organization are critical levers for enhancing system sustainability. Ultimately, tailored policy support and inclusive planning will be necessary to maintain the viability of pastoral livelihoods in the face of environmental and socio-economic change.

## Figures and Tables

**Table 1:** Variables describing the structure and management of farms included in the data analysis.

Category	Variable (Name)	Type	Unit or Modalities	Description	Summary Statistics
General farm characteristic	Zone	Categorical	Dhahar, Urban, Waara	Waara 61%, Dhahar 25%, Urban 14%	
	Academic level	Categorical	Illiterate, Undergraduate, Graduate	Highest educational level of the farmer	Undergraduate 60%, Illiterate 31%, Graduate 9%
	Farming main activity	Categorical	Yes/No	Whether livestock is the main activity	No 58%, Yes 42%
	Workforce	Categorical	Family, Contracted	Type of labor used on the farm	Family 66%, Contracted 34%
Flock management	Handling	Categorical	Family, Contracted, Mixed	Responsible for flock management	Family 47%, Contracted 44%, Mixed 9%
	Number of handlings	Numerical	People	Number of people handling the animals	2.31 ± 1.11
	Type of goats sold	Categorical	Young, Males and Culls, Need	Category of goats sold	Young 58.6%, Need 32.4%, Males and Culls 9%
	Reason for sale	Categorical	Commercial, Habit, Necessity	Reason for selling animals	Necessity 59.5%, Habit 21.6%, Commercial 18.9%
	Date of sale	Categorical	Availability, Seasonal	Timing of animal sale	Availability 72%, Seasonal 28%
	Type of sheep sold	Categorical	Young, All	Category of sheep sold	Young 64%, All 36%
Flock size and structure	Total goats	Numerical	Goats	Total number of goats in the flock	86.75 ± 64.80
	Goat sex ratio	Numerical	Ratio	Male-to-female ratio in goat flock	4.72 ± 2.15
	Total sheep	Numerical	Sheep	Total number of sheep in the flock	319.66 ± 218.79
	Sheep sex ratio	Numerical	Ratio	Male-to-female ratio in sheep flock	4.25 ± 1.97
	Total camels	Numerical	Camels	Total number of camels	10.92 ± 35.91
	Goat breed composition	Categorical	Pure, Mixed	Breed composition of goats	Pure 78%, Mixed 22%
Natural resources and infrastructure	Water source	Categorical	Collective, Private	Source of water for the animals	Collective 67%, Private 33%
	Distance to water	Categorical	<10 km, >10 km	Distance from farm to water source	<10 km 72%, >10 km 28%
	Water problems	Categorical	Yes, No	Occurrence of problems related to water supply	No 64%, Yes 36%
	Infrastructure	Categorical	Fixed, Mobile, Building	Type of physical infrastructure available	Fixed 54%, Mobile 30%, Building 16%
Feeding practices	Separation of feed	Categorical	Yes, No	Whether feeding is separated by species	No 25%, Yes 75%
	Rangeland property	Categorical	Collective, Private	Ownership type of grazing land	Collective 75%, Private 25%
	Olive pomace use	Categorical	Yes, No	Use of olive pomace in animal feeding	No 73%, Yes 27%
	Bran use	Categorical	Yes, No	Use of bran in feeding	Yes 70%, No 30%
	Concentrate use	Categorical	Yes, No	Use of concentrate feed	No 84%, Yes 16%
	Hay use	Categorical	Yes, No	Use of hay in feeding	No 80%, Yes 20%
	Straw use	Categorical	Yes, No	Use of straw in feeding	No 65%, Yes 35%
Reproduction and health	Reproduction strategy	Categorical	Controlled, Uncontrolled	Type of breeding strategy	Uncontrolled 61%, Controlled 39%
	Sheep male efficiency	Categorical	Yes, No	Effective use of breeding males in sheep	No 54%, Yes 46%
	Goat male efficiency	Categorical	Yes, No	Effective use of breeding males in goats	No 74%, Yes 26%
	Prophylactic plan	Categorical	Yes, No	Use of preventive health measures	No 34% Yes 66%,
	Weaning	Categorical	Yes, No	Whether weaning is practiced	No 51%, Yes 49%

Note: Sheep breed was not included as a variable because the Barbarine breed represents the overwhelming majority (>95%) of the livestock in all sampled farms.

**Table 2:** Contribution of Selected Variables to the Principal Dimensions (FAMD).

Variable	Dimension 1	Dimension 2	Dimension 3
Total number of goats	0.70	0.10	0.08
Total number of sheep	0.60	0.12	0.09
Number of camels	0.39	0.15	0.12
Distance to water source	0.25	0.44	0.20
Availability of infrastructure	0.20	0.35	0.15
Use of feed supplements	0.18	0.31	0.12
Reproductive strategy	0.10	0.22	0.49
Water problem index	0.15	0.28	0.36
Workforce	0.10	0.21	0.27
Handling	0.12	0.18	0.22
Number of handlings	0.18	0.20	0.18
Transhumance	0.22	0.22	0.20
Goat-type animal sold	0.15	0.19	0.19
Reason sold	0.20	0.21	0.18
Date of sale	0.25	0.24	0.20
Ovine-type animal sold	0.19	0.18	0.15
Mixed breed goat	0.14	0.17	0.12
Water source	0.22	0.25	0.18
Separation feed	0.18	0.20	0.14
Property rangeland	0.15	0.18	0.12
Olive pomace	0.12	0.15	0.10
Bran	0.10	0.14	0.09
Concentrate	0.14	0.12	0.11
Hay	0.10	0.10	0.12
Straw	0.08	0.09	0.10
Ovine male effect	0.09	0.11	0.08
Goat male effect	0.12	0.12	0.10
Weaning	0.15	0.14	0.12
Prophylactic plan	0.14	0.10	0.14

**Table 3:** Percentage of responses for the categorical variables within each cluster and statistical differences between them.

Variable Name	Type 1	Type 2	Type 3	Significance
Farming main activity (Yes)	54.3%	44.7%	28.9%	**
Type of handling (Family labor)	71.4%	23.7%	47.4%	**
Type of handling (Hired labor)	28.6%	71.1%	31.6%	**
Type of handling (Both familial and contracted)	0.0%	5.3%	21.1%	**
Type of seasonal workforce (Familial)	80%	50%	79%	**
Type of seasonal workforce (Contracted)	20%	50%	21%	**
Property water source (Communal well)	20%	82%	95%	****
Property water source (Private)	80%	18%	5%	****
Distance to the water source (<10 km)	83%	74%	61%	***
Distance to the water source (>10 km)	17%	26%	39%	***
Existence of water problems (Yes)	54%	24%	29%	***
Types of farm infrastructure (Building)	0%	5%	39%	***
Types of farm infrastructure (Fixed)	74%	63%	26%	***
Types of farm infrastructure (Mobile)	26%	32%	34%	ns
Feed is separated for different animals (Yes)	60%	71%	92%	***
Ownership status of rangeland (Collective)	40%	84%	97%	****
Ownership status of rangeland (Private)	60%	16%	3%	****
Use of olive pomace in feeding (Yes)	69%	5%	11%	****
Use of bran in feeding (Yes)	97%	71%	45%	****
Use of concentrate in feeding (Yes)	37%	3%	11%	****
Use of hay in feeding (Yes)	60%	3%	0%	****
Use of straw in feeding (Yes)	60%	3%	0%	****
Breeding strategy (Controlled)	23%	26%	68%	***
Breeding strategy (Uncontrolled)	77%	74%	32%	***
Practice of male effect in reproduction (Yes)	17%	39%	74%	***
Male effect in goats (Yes)	17%	11%	47%	***
Weaning practices on the farm (Yes)	66%	24%	53%	***
Prophylactic health plan (Yes)	77%	79%	42%	***
Practices of transhumance (Yes)	43%	71%	87%	***
Goat breed composition (Mixed)	46%	16%	5%	****
Goat breed composition (Pure)	54%	84%	95%	****
Types of goats that are sold (Need)	26%	53%	18%	***
Types of goats that are sold (Male + culls)	17%	11%	0%	***
Types of goats that are sold (All young animals)	57%	37%	82%	***
Reason for selling animals (Necessity)	11%	34%	18%	***
Reason for selling animals (Habitual)	31%	5%	21%	***
Reason for selling animals (Commercial)	57%	61%	61%	ns
Timing of animal sales (Availability)	74%	55%	92%	**
Timing of animal sales (Seasonal)	26%	45%	8%	**
Types of sheep sold (All young animals)	29%	34%	42%	*
Types of sheep sold (As needed)	71%	66%	58%	*

Significance levels: ns = not significant; *p* < 0.05 (*), *p* < 0.01 (**), *p* < 0.001 (***), *p* < 0.0001 (****).

**Table 4:** Mean values of numerical variables across the three pastoral farming systems, with global averages and significance of differences between systems.

Cluster Name	Small Urban Farmer	Large Farms	Large Farms with Camel	Global Average	Significance
Number of handlings	2	2	3	2.3	**
Total number of sheep	171	330	446	319	***
Total number of goats	49	126	83	87	**
Number of camels	3	6	23	11	***

Significance levels: *p* < 0.01 (**), *p* < 0.001 (***).

## Data Availability

The data supporting the findings of this study are available from the corresponding author upon reasonable request.
